# Bacterial Signatures of Cerebral Thrombi in Large Vessel Occlusion Stroke

**DOI:** 10.1128/mbio.01085-22

**Published:** 2022-06-21

**Authors:** Yu Liao, Xiuli Zeng, Xiaomei Xie, Dan Liang, Hongyu Qiao, Wence Wang, Min Guan, Shengming Huang, Zhen Jing, Xinyi Leng, Li’an Huang

**Affiliations:** a Department of Neurology, The First Affiliated Hospital, Jinan University, Guangzhou, China; b Department of Pathology, The First Affiliated Hospital, Jinan University, Guangzhou, China; c Guangdong Provincial Key Laboratory of Animal Nutrition and Regulation, College of Animal Science, South China Agricultural University, Guangzhou, China; d Department of Medicine & Therapeutics, The Chinese University of Hong Kong, Hong Kong SAR, China; Brigham and Women's Hospital

**Keywords:** bacteria, acute ischemic stroke, mechanical thrombectomy, 16S sequencing, FISH

## Abstract

It is important to understand the microbial features of the cerebral thrombus and its clinical relevance in stroke patients, of which data were scarce. We aimed to investigate the microbial features of cerebral thrombi retrieved via thrombectomy in stroke patients with large vessel occlusion (LVO) and their correlations with 3-month mortality. In a prospective cohort study, thrombus samples were collected during mechanical thrombectomy in LVO stroke patients with successful revascularization at a tertiary hospital. Oral, fecal, and isolated plasma samples were collected within 12 h of admission. The microbial compositions of all samples were compared using 16S rRNA gene amplicon next-generation sequencing. Fluorescent *in situ* hybridization (FISH) was used to detect bacteria in thrombus samples. The primary outcome was 3-month mortality. Perioperative adverse events (AEs) within 48 h were also recorded. Bacterial DNA was detected in 96.2% of thrombus samples from 104 patients, and clusters of bacterial signals were seen in the thrombi with FISH. Compared with fecal and oral samples, the thrombus microbiota was mainly characterized by excessive enrichment of *Proteobacteria*, mainly originating from plasma. The bacterial concentrations, dominant bacteria, and distribution patterns differed in thrombi obtained from cardioembolic and large-artery atherosclerotic strokes. Higher abundances of Acinetobacter and *Enterobacteriaceae* were associated with a higher risk of perioperative AEs, and a higher abundance of Acinetobacter was independently associated with a higher risk of 90-day mortality. This study demonstrated the presence of bacteria in cerebral thrombi retrieved with thrombectomy in LVO strokes, with some bacteria associated with patients’ prognoses.

## INTRODUCTION

There have been emerging research efforts on the brain-gut axis, i.e., the interaction between stroke and the gut microbiota (1–5), indicating close and reciprocal relationships of the commensal microbiota with thrombin generation, arterial thrombosis, and stroke (3, 6–8). For instance, Toll-like receptor 2 (TLR2) signaling induced by the microbiota stimulates von Willebrand factor (VWF) synthesis in the hepatic endothelium of mice, promoting thrombus growth in the ligation-injured carotid artery (9). Gut microorganisms could also directly increase platelet hyperreactivity and accelerate thrombosis by producing trimethylamine *N*-oxide (TMAO), a common bacterial metabolite (10). Moreover, after an acute stroke, the hyperpermeability of the intestinal barrier enhances translocation and transmission of selective strains from the host intestinal microbiota (e.g., microbiome dysbiosis) (11), which, in turn, exacerbates cerebral infarction and the subsequent outcomes (3).

Recent studies have also revealed that microbiota colonization in thrombi of patients with myocardial infarction might influence the progression of thrombosis and risks of major adverse cardiovascular events (12–15). Yet data were scarce on microbial analysis of thrombi from stroke patients before endovascular mechanical thrombectomy was established as a first-line treatment for acute ischemic stroke (AIS) with large vessel occlusion (LVO) (16–21). Endovascular treatment not only significantly improves outcomes of LVO stroke patients compared with conventional treatment but also provides a unique opportunity for investigations of the thrombus in such patients, including the thrombus microbiota (22–24). However, some recent relevant studies had conflicting findings on the presence of bacteria in retrieved cerebral thrombi in such patients (22–24), and little is known regarding the source of the thrombus microbiota and its association with outcomes after thrombectomy. In addition, these studies mostly had a small sample size or simplified methodology on microbiota analysis (e.g., quantitative PCR [qPCR] amplification).

In this study, we aimed to investigate the microbial features of cerebral thrombi collected by stent retriever within 24 h of onset in LVO stroke patients, using advanced sequencing technology (16S rRNA gene amplicon next-generation sequencing), fluorescent *in situ* hybridization (FISH), and fast expectation-maximization microbial source tracking (FEAST), and to correlate the microbial features with postthrombectomy outcomes.

## RESULTS

### Patient characteristics.

We recruited 104 AIS patients (median age, 65 years) who underwent thrombectomy, with 60 (57.7%) males. The median onset-to-arrival time was 360 minutes (interquartile range [IQR], 231 to 592), and the median onset-to-reperfusion time was 455 minutes (IQR, 292 to 628). The median preoperative National Institute of Health Stroke Scale (NIHSS) score was 17 (IQR, 13 to 20). The stroke etiology was large-artery atherosclerosis (LAA) in 59 (56.7%) patients and cardioembolism (CE) in the remaining patients. None of the patients showed signs of gingival bleeding or acute periodontal disease. Other characteristics of the patients are shown in Table 1.

**TABLE 1:** Characteristics of enrolled patients (*n* = 104)

Characteristic^*a*^	Value
Age (median [IQR] [yrs])	65 (58–75)
Male sex (no. [%])	60 (57.7)
Ever smoking (no. [%])	41 (39.4)
Alcohol abuse (no. [%])	16 (15.4)
Medical history	
Prior stroke (no. [%])	21 (20.2)
Diabetes mellitus (no. [%])	35 (33.7)
Hypertension (no. [%])	71 (33.7)
Atrial fibrillation (no. [%])	39 (37.5)
Heart disease^*b*^ (no. [%])	35 (33.7)
Occluded artery (no. [%])	
ICA	41 (39.4)
MCA	40 (38.5)
BA	21 (20.2)
TOAST classification (no. [%])	
Cardioembolism	45 (43.3)
Large-artery atherosclerosis	59 (56.7)
Preoperative NIHSS score (median [IQR])	17 (13–20)
Onset-to-arrival time (median [IQR] [min])	360 (231–592)
Onset-to-reperfusion time (median [IQR] [min])	455 (292–628)
Prior intravenous thrombolysis (no. [%])	27 (26.0)
Hospital stay (median [IQR] [days])	12 (8–15)
Discharged NIHSS score (median [IQR])	8 (3–12)
Perioperative adverse events (no. [%])	39 (37.5)
Poststroke infection (no. [%])	57 (54.8)
90-day outcomes	
90-day mRS score (median [IQR])	3 (1–4)
90-day mortality (no. [%])	17 (16.3)

a NIHSS, National Institute of Health Stroke Scale; ICA, internal carotid artery; MCA, middle cerebral artery; BA, basilar artery; TOAST, Trial of Org 10172 in Acute Stroke Treatment; mRS, modified Rankin scale.

b Heart disease refers to coronary artery disease, valvular heart disease, and congestive heart failure.

### Microbial features of thrombus, plasma, fecal, and oral samples.

**FIG 1:**
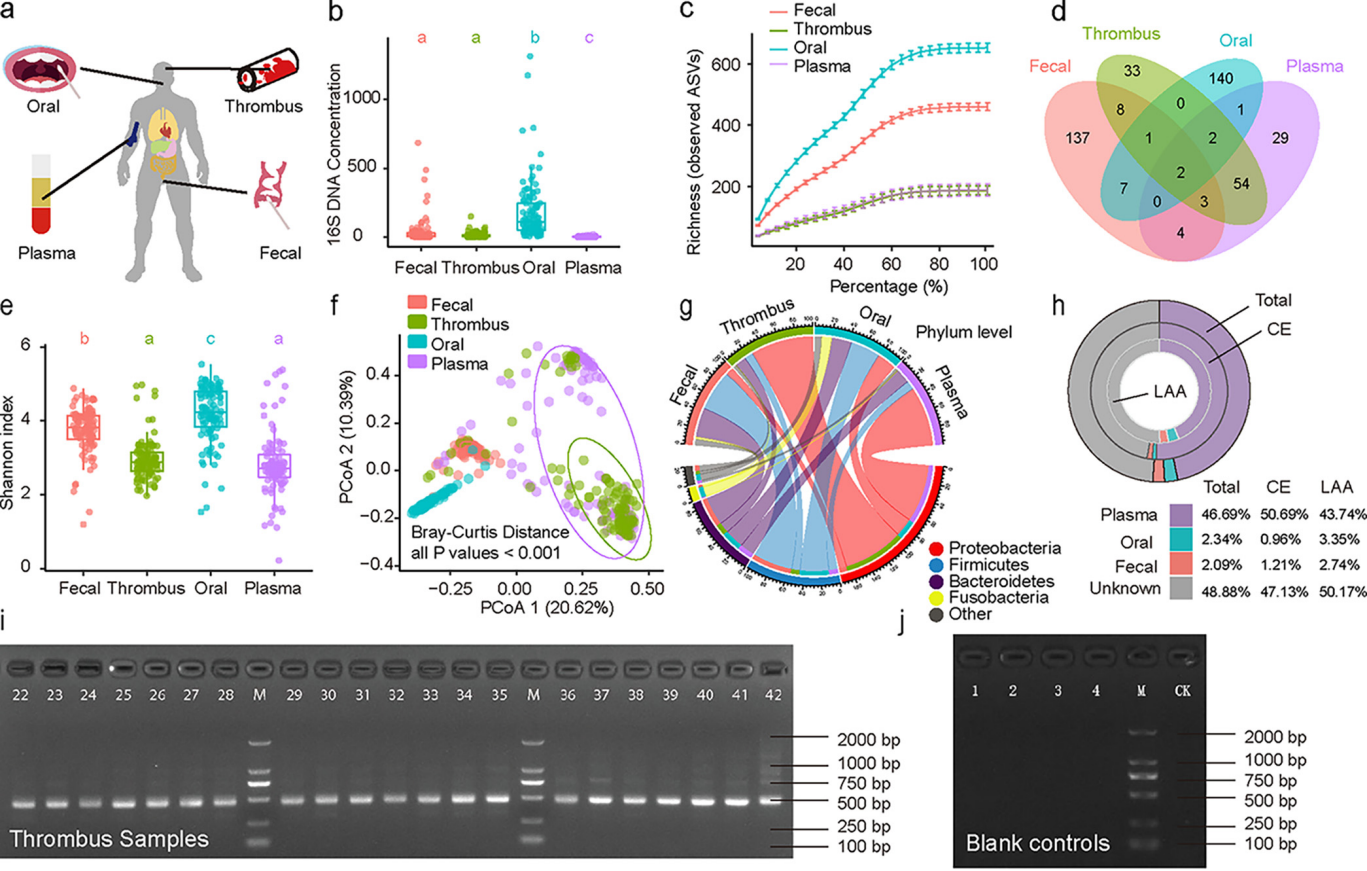
Bacterial composition of the different communities among the four body habitats. (a) Schematic diagram of the body sites of the bacterial communities in the present study. (b) Concentration of bacterial DNA in different samples. Lowercase letters a, b, and c are used to show differences between compared groups; different letters represent significant differences between the two groups, while the same letters represent no difference. Each dot denotes a sample. (c) Rarefaction curves of detected bacterial ASVs among the four types of samples reaching saturation with increasing sequencing depths. The vertical bars represent standard errors. (d) Venn diagram representing the overlapping ASVs in the four types of samples (only ASVs with a relative abundance greater than 0.1% are shown in the diagram). (e) Shannon indices of the four types of samples. Lowercase letters a, b, and c are used to show differences between compared groups as in panel B. Each dot denotes a sample. (f) Microbial composition of thrombus samples was obviously distinct from that of fecal, oral, and plasma samples, with the Bray-Curtis distance scored on the relative abundance of ASVs. (g) Relative abundances of the dominant taxa in the four types of samples at the phylum level. (h) Source tracking of the microbiota in the thrombi based on the FEAST algorithm in all patients and in patients with CE and LAA stroke, respectively. (i) PCR products of thrombus samples, with 470-bp bands as target products. M, marker DL2000; CK, reagent control in the PCR process. (j) No targeted PCR products were observed in the blank control samples from collection tubes. M, marker DL2000; CK, reagent control in the PCR process. Abbreviations: ASV, amplicon sequence variant; FEAST, fast expectation-maximization for microbial source tracking; CE, cardioembolism; LAA, large-artery atherosclerosis; PCR, polymerase chain reaction.

Overall, we collected 104 thrombus, 98 plasma, 103 fecal, and 102 oral samples from the 104 patients recruited. Bacterial DNA was positive in 96.2% (100/104) of the thrombus samples (Fig. 1i) and 96.9% (95/98) of the isolated plasma samples in qPCR. Blank control samples obtained from collection tubes (Fig. 1j) and operation table (e.g., surgical drape, skin at the puncture point, interventional instruments, the syringe to transfer the thrombus, and interventionalist's gloves) were bacteria negative in qPCR (see Fig. S1 in the supplemental material). Also, reagent controls (including all qPCR reagents such as sterile water and primers, but without DNA templates) were contained in all qPCR experiments, and no targeted PCR products were observed (Fig. S2 and S3). Thus, exogenous contamination (e.g., surgical and sample disposal and analysis procedures) of bacteria can be ruled out.

10.1128/mbio.01085-22.2 FIG S1 Gel electrophoresis result of plate 1 for blank controls. (a and b) Numbers 1 to 38 represent periprocedural environmental controls as follows. Before operation, surgical drape, 1 to 4; surgical gloves, 5 to 8. During operation, arterial blood on the puncture needle, 9 to 12; skin of the puncture point, 13 to 16; intervention instrument, 21 to 24. After operation, surgical gloves, 17 to 20; arterial blood on the puncture point, 35 to 38. Collection and transferal of thrombus, etc., external surface of saline washed thrombus, 25 to 26; syringe to transfer the thrombus, 27 to 30; Eppendorf tube for thrombus collection, 31 to 34. “M” represents marker, also shown in a larger view, with the target amplified fragment in the V3V4 region of 16S rRNA with a fragment size of 470 bp. CK, reagent control (without DNA template) for polymerase chain reaction. Download FIG S1, TIF file, 1.9 MB. Copyright © 2022 Liao et al. 2022 Liao et al. https://creativecommons.org/licenses/by/4.0/ This content is distributed under the terms of the Creative Commons Attribution 4.0 International license.

10.1128/mbio.01085-22.3 FIG S2 Gel electrophoresis result of 7 plates for collected samples. (1a to 7b) Gel electrophoresis results of 7 plates of various samples collected in this study, each with a marker (M) and reagent control (without DNA template) for polymerase chain reaction (CK). The numbers in the plates represent different samples that are sorted randomly. Download FIG S2, TIF file, 2.0 MB. Copyright © 2022 Liao et al. 2022 Liao et al. https://creativecommons.org/licenses/by/4.0/ This content is distributed under the terms of the Creative Commons Attribution 4.0 International license.

10.1128/mbio.01085-22.4 FIG S3 Gel electrophoresis result of another 7 plates for collected samples. (1a to 7a) Gel electrophoresis results of another 7 plates of various samples collected in this study, each with a marker (M) and reagent control (without DNA template) for polymerase chain reaction (CK). The numbers in the plates represent different samples that are sorted randomly. Download FIG S3, TIF file, 1.9 MB. Copyright © 2022 Liao et al. 2022 Liao et al. https://creativecommons.org/licenses/by/4.0/ This content is distributed under the terms of the Creative Commons Attribution 4.0 International license.

Microbiota from 100 thrombus, 95 plasma, 103 fecal, and 102 oral samples was characterized by 16S rRNA amplicon sequencing. The median 16S rRNA concentration of thrombus (9.23 ng/μL) samples was lower than that of oral (109.69 ng/μL) and fecal (12.61 ng/μL) samples but higher than that of plasma (3.32 ng/μL) samples (Fig. 1b). The rarefaction curves show that the amplicon sequence variant (ASV) numbers in the thrombus and plasma samples were similar, but both were lower than those in the fecal and oral samples (Fig. 1c). In the Venn diagram, there were 70 highly abundant ASVs in the thrombus samples that were shared with bacteria from other sites. The number of unique sequences was smallest in the thrombus plasma samples (29) and largest in the oral samples (140) (Fig. 1d). In terms of alpha diversity, the Shannon index of the thrombus samples significantly differed from that of the fecal and oral samples (all *P* < 0.05) but was similar to that of the plasma samples (Fig. 1e). The microbial composition of thrombus samples was obviously distinct from that of fecal, oral, and plasma samples by the Bray-Curtis distance analysis (Adonis test, all *P* < 0.001) (Fig. 1f).

There were 14 phyla, 182 genera, and 542 ASVs that had a relative abundance >1.0% across thrombus samples. The top 20 relative abundances of genera in thrombi were Acinetobacter (12.8%), *Burkholderia* (12.5%), *Sphingomonas* (10.9%), *Pedobacter* (7.0%), *Serratia* (5.5%), *Stenotrophomonas* (5.3%), *Brevundimonas* (3.8%), *Bradyrhizobium* (3.1%), *Bacillus* (2.1%), *Elizabethkingia* (1.6%), *Prevotella* (1.6%), *Ochrobactrum* (1.5%), *Ralstonia* (1.4%), *Herbaspirillum* (1.3%), *Chryseobacterium* (1.3%), *Methylobacterium* (1.3%), Staphylococcus (1.2%), *Enhydrobacter* (1.1%), Streptococcus (1.1%), and *Clostridium_sensu_stricto* (1%). At the phylum level (Fig. 1g), most bacteria in thrombus samples belonged to the phyla *Proteobacteria* (relative abundance, 73.3%, including 12 of the top 20 genera above), *Bacteroidetes* (12.9%), and *Firmicutes* (10.0%) and less to *Actinobacteria* (2.0%). In general, the bacterial composition of the thrombus samples was similar to that of the plasma samples but distinct from that of the oral and fecal samples.

Bacterial culture was positive in 2 out of 8 thrombus aspirates, which were identified as Bacillus cereus, Lactobacillus vaginalis, and Kocuria marina, all of which were detected in our sequencing data cohort.

### Source tracking of thrombus microbiota and function prediction by BugBase.

**FIG 2:**
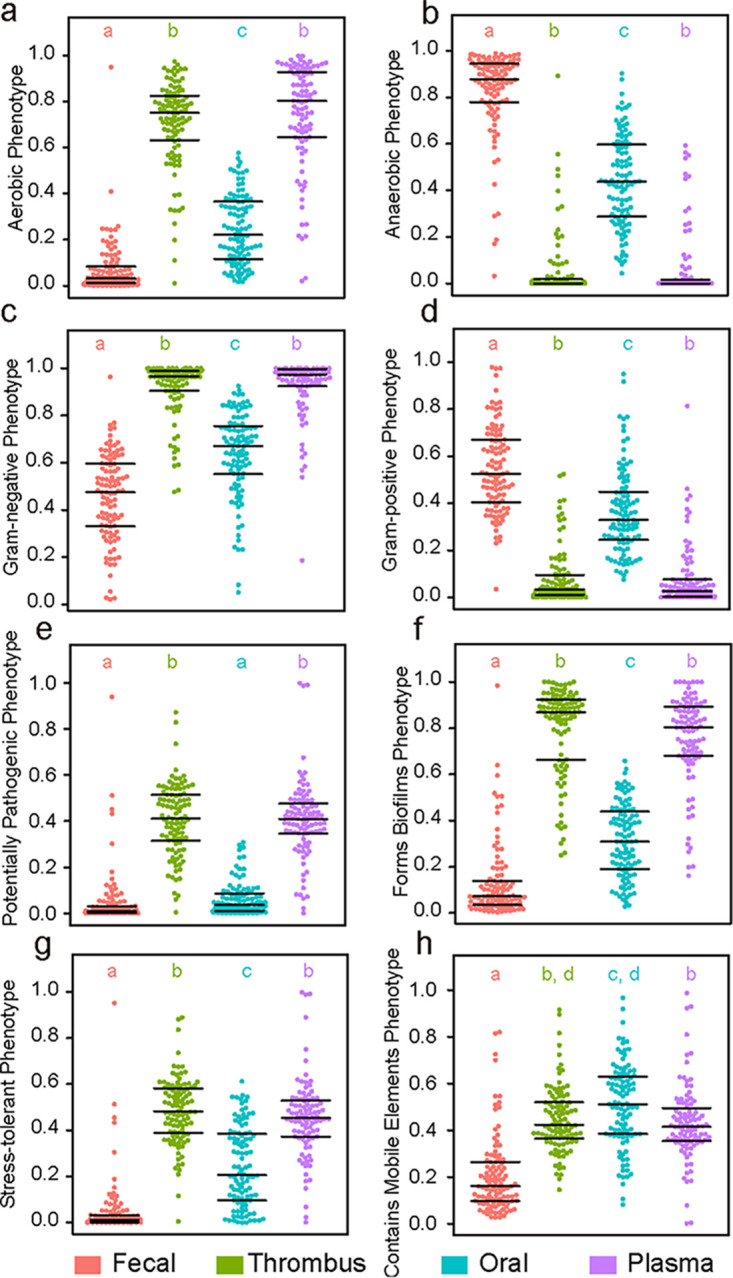
Bacterial compositions in the four types of samples predicted by BugBase. Lowercase letters a, b, c, and d are used to represent differences between groups with Bonferroni correction following Kruskal-Wallis tests: different letters represent significant difference between two groups, while the same letters represent no difference.

The thrombus microbial community had approximately 46.7% of the community sourced from plasma, only 2.3% from oral, and 2.1% from fecal samples (Fig. 1h); the matching proportions of ASVs from different sources for each thrombus sample are shown in Table S1. We observed a significantly higher representation of aerobic bacteria, Gram-negative bacteria, potentially pathogenic bacteria, bacteria related to biofilm formation, and oxidative stress-tolerant bacteria in the thrombus and plasma samples (Fig. 2). Regarding the etiology of the index LVO stroke, the thrombus bacteria in CE patients tended to be more likely to derive from plasma (50.7% versus 43.7%; *P* = 0.122) than in LAA patients, but without statistical difference.

10.1128/mbio.01085-22.1 TABLE S1 Proportions of bacteria in thrombus samples from different sources in each patient. Download Table S1, XLSX file, 0.02 MB. Copyright © 2022 Liao et al. 2022 Liao et al. https://creativecommons.org/licenses/by/4.0/ This content is distributed under the terms of the Creative Commons Attribution 4.0 International license.

### Signature of thrombus microbiota via FISH.

**FIG 3:**
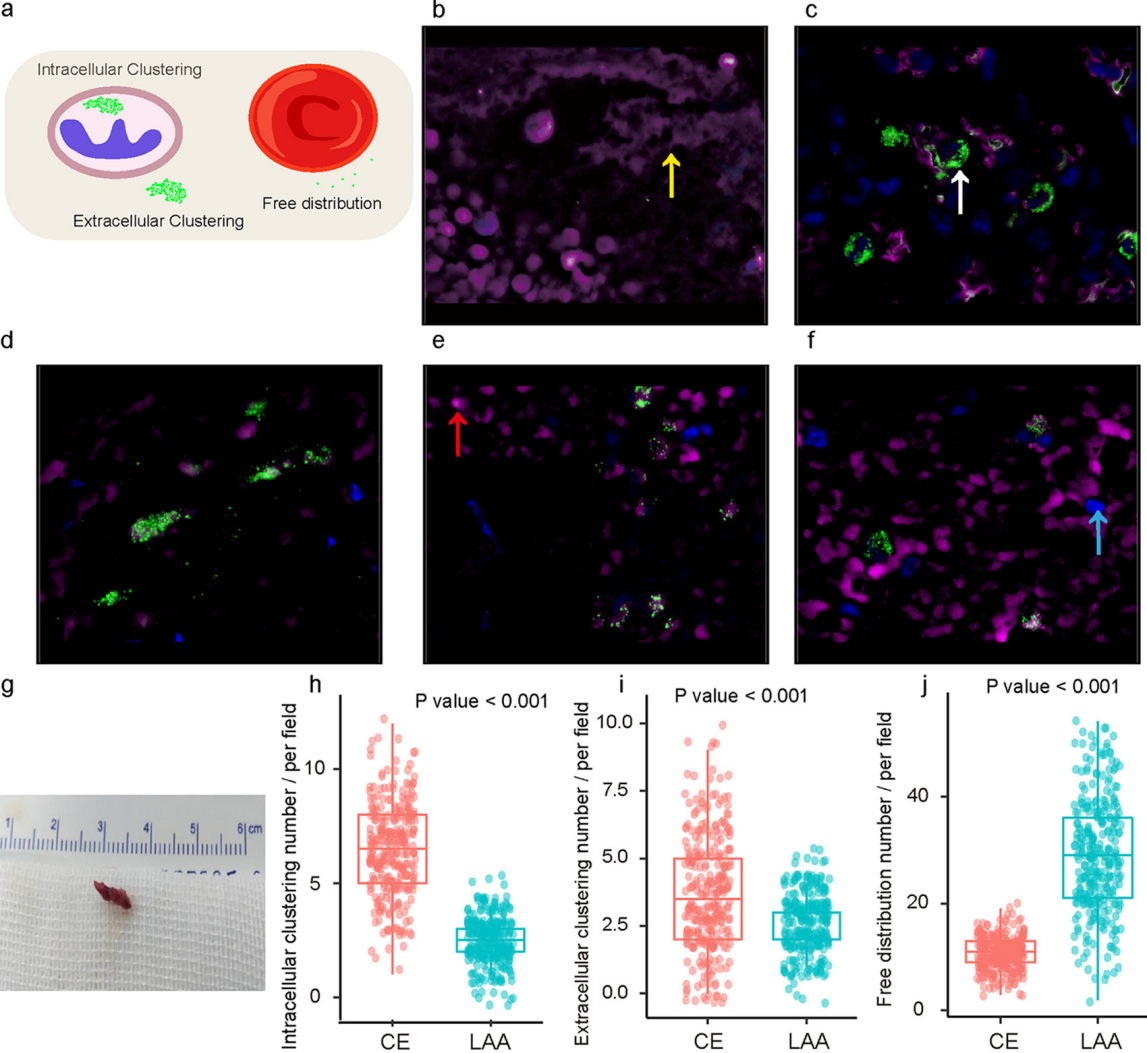
Bacterial signature in the thrombus samples via FISH. (a) Classical representation of bacterial distribution patterns. (b to f) General compositions of thrombi in FISH visualized by fluorescence microscopy (magnification, ×1,000). Green fluorescence signals represent bacteria in the thrombus (white arrow), purple-red irregular round signals are red blood cells (red arrow), sheet of purple briquette is fibrin (yellow arrow), and the blue shapes are DAPI-stained white blood cells or macrophages (blue arrow). Among them, panels c to d and e to f, respectively, show typical bacterial distribution patterns in patients with cardioembolic stroke and large-artery atherosclerotic stroke. (g) Classic appearance of a thrombus retrieved from a stroke patient by mechanical thrombectomy. (h to j) Comparisons of bacteria distribution patterns in thrombi from CE versus LAA stroke patients. Each dot represents the count of bacteria-positive signals in each field (10 fields were randomly selected in each thrombus sample for visual counting of bacteria-positive signals). Abbreviations: FISH, fluorescence *in situ* hybridization; DAPI, 4,6-diamidino-2-phenylindole; CE, cardioembolism; LAA, large-artery atherosclerosis.

FISH of thrombus sections was performed in 58 AIS patients (50.0% with CE and 50.0% with LAA stroke) with adequate thrombus tissue (e.g., the thrombus in Fig. 3g). As described previously (25), the main components of thrombi in stroke patients are red blood cells, white blood cells, fibrin, and aggregated platelets. The red blood cells, fibrin, and aggregated platelets were visualized in a purplish red color in FISH slices (Fig. 3b), and nucleated cells such as neutrophils and macrophages were visualized in blue color (Fig. 3f). Green dot fluorescence in thrombus samples was a positive signature of bacteria (Fig. 3c). Ten visual fields with obvious bacteria-positive signals in each thrombus sample were randomly selected, which revealed 3 major patterns of microbiota distribution in thrombus, free distribution, intracellular clustering (single green signal dot > 5), and extracellular clustering. The distribution patterns were significantly different between patients with CE and LAA strokes (Fig. 3h to j). The thrombi in CE patients were characterized as a clustered microbial distribution pattern with intracellular and extracellular clustering (Fig. 3c to 3d), while LAA patients had mainly free microbial distributions (Fig. 3e to 3f).

### Thrombus microbial peculiarity in CE versus LAA strokes.

**FIG 4:**
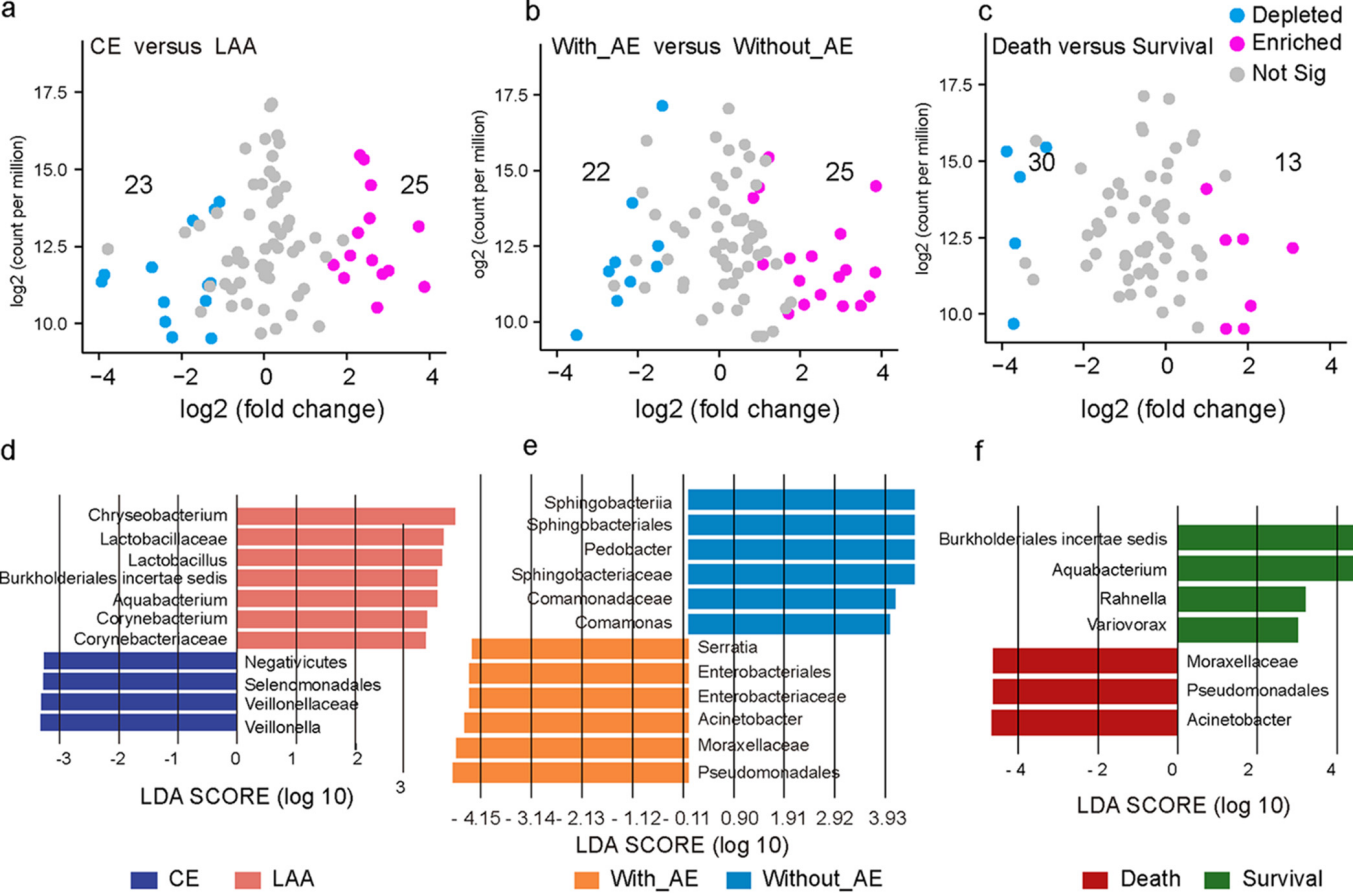
Microbial characteristics of thrombi by stroke subtypes and clinical outcomes. (a to c) Numbers of differentially enriched ASVs with an average abundance higher than 0.1% between two groups with FDRs of <0.05 are presented in volcano plots. Each point represents an individual ASV, and the position along the *y* axis represents the log_2_ value of average abundance. (d to f) Significantly discriminative taxa between two groups were determined using linear discriminant analysis and effect size analysis. Abbreviations: FDR, false-discovery rate; CE, cardioembolism; LAA, large-artery atherosclerosis; AE, adverse event. ASV, amplicon sequence variant; LDA, linear discriminant analysis.

The 16S rRNA concentrations in thrombus samples from patients with CE stroke were remarkably higher than those with LAA stroke (medians, 11.35 versus 7.22 ng/μL; *P* = 0.007). For specific ASVs, 23 ASVs were significantly depleted and 25 enriched in thrombus samples from CE strokes compared with LAA strokes (Fig. 4a). Linear discriminant analysis (LDA) coupled with effect size measurement (LEfSe) revealed different dominant bacteria in thrombi obtained from CE and LAA stroke patients (Fig. 4d), the *Veillonellaceae* family in the phylum *Firmicutes* in CE stroke, but *Chryseobacterium* and *Lactobacillaceae* families in LAA stroke.

### Thrombus microbiota and other variables associated with perioperative.

Thirty-nine (37.5%) patients had perioperative AEs, including 2 patients with new neurological symptoms accompanied by new ischemic lesions in imaging, 34 patients with hemorrhagic transformation (13 with hemorrhagic infarction and 21 with parenchymal hematoma), and 9 patients with cerebral herniation. Enzyme-linked immunosorbent assay (ELISA) of plasma isolated within 12 h after admission showed significantly higher concentrations of interleukin-1β (IL-1β) (*P* = 0.004) and IL-6 (*P* = 0.002) in the plasma of patients with perioperative AEs than those without. Plasma markers of intestinal barrier integrity were not significantly different between the two groups (“with AE” and “without AE” groups, Table 2). As shown by the Volcano plot in Fig. 4b, 47 ASVs were significantly different in thrombus samples from those with and without perioperative AE, with a false-discovery rate (FDR) of <0.05. Further LEfSe analysis revealed that Acinetobacter (order, *Pseudomonadales*; family, *Moraxellaceae*) and *Enterobacteriaceae* were enriched in cerebral thrombi in the With_AE group (Fig. 4e).

**TABLE 2:** Comparisons in markers of inflammation and intestinal barrier integrity in patients with and without perioperative AE^*a*^

Marker	With AE group (*n* = 35)	Without AE group (*n* = 60)	*P* value
WBC (10^9^/L)	10.87 (8.74–12.80)	9.83 (7.91–12.33)	0.352
CRP (mg/L)	9.67 (3.48–26.08)	7.85 (3.16–15.69)	0.547
IL-1β (pg/mL)	41.17 (27.47–75.33)	29.56 (17.78–47.39)	0.004
IL-6 (pg/mL)	14.58 (3.79–35.91)	3.30 (0–12.25)	0.002
TNF-α (pg/mL)	48.77 (42.67–58.32)	46.55 (37.06–57.22)	0.371
LPS (pg/mL)	49.25 (39.85–58.38)	48.04 (32.17–71.83)	0.963
LBP (μg/mL)	5.21 (4.01–5.77)	4.78 (3.40–5.95)	0.357
d-Lactate (mmol/L)	10.67 (7.32–13.35)	9.09 (7.62–12.04)	0.343

a Data are presented in medians (interquartile range). *P* values were determined by Mann-Whitney U tests. AE, adverse event; WBC, white blood cells; CRP, C-reactive protein; LPS, lipopolysaccharide; LBP, lipopolysaccharide-binding proteins.

There was no significant association of oral or fecal microbiota with the presence of perioperative AE (Fig. S4b and d). However, higher abundances of *Paracoccus* and Streptococcus were observed in the plasma samples of patients with perioperative AE (Fig. S5b).

10.1128/mbio.01085-22.5 FIG S4 Comparisons of oral/fecal microbial compositions in patients with or without the clinical outcomes. (a) Abundance of dominant bacteria in different samples from each patient at the genus level. (b) Comparison of dominant oral bacteria at the genus level between those with and without perioperative AEs. (c) Comparison of dominant oral bacteria at the genus level between those who survived and died within 90 days. (d) Comparison of dominant fecal bacteria at the genus level between those with and without perioperative AEs. (e) Comparison of dominant fecal bacteria at the genus level between those who survived and died within 90 days. Abbreviation: AE, adverse event. Download FIG S4, TIF file, 0.9 MB. Copyright © 2022 Liao et al. 2022 Liao et al. https://creativecommons.org/licenses/by/4.0/ This content is distributed under the terms of the Creative Commons Attribution 4.0 International license.

10.1128/mbio.01085-22.6 FIG S5 Comparisons of plasma microbiome in patients with or without perioperative AEs (a to c) and in those who survived and died within 90 days (d to f). (a) Numbers of differentially enriched ASVs with an average abundance higher than 0.1% between those with and without perioperative AEs, with an FDR of <0.05, are presented in volcano plots. Each point represents an individual ASV, and the position along the *y* axis represents the log_2_ value of average abundance. Forty-four ASVs were significantly different in those with and without perioperative AE. (b) Significantly discriminative taxa between the two groups were determined using LefSe. Increased plasma *Paracoccus* and Streptococcus were observed in patients with perioperative AE. (c) Numbers of differentially enriched ASVs with an average abundance higher than 0.1% between those with an FDR of <0.05 who survived and died within 90 days are presented in volcano plots. Each point represents an individual ASV, and the position along the *y* axis represents the log_2_ value of average abundance. Fifty-six ASVs were significantly different in those who survived and died within 90 days. (d) Significantly discriminative taxa between the two groups were determined using LefSe. Increased plasma Acinetobacter and *Paracoccus* and decreased *Bacteroidetes* were observed in those patients died within 90 days. Abbreviations: FDR, false-discovery rate; AE, adverse event; ASV, amplicon sequence variant; LDA, linear discriminant analysis. Download FIG S5, TIF file, 0.8 MB. Copyright © 2022 Liao et al. 2022 Liao et al. https://creativecommons.org/licenses/by/4.0/ This content is distributed under the terms of the Creative Commons Attribution 4.0 International license.

### Thrombus microbiota and other variables associated with 90-day mortality.

During 90 days of follow-up, 17 (16.3%) patients died, and they had higher white blood cell levels at admission than those who survived at 90 days (*P* = 0.025). ELISA of plasma revealed significantly higher levels of IL-1β (*P* = 0.001) and IL-6 (*P* = 0.001) at baseline in stroke patients who later died (Table 3). These findings suggested possibly more severe systemic inflammation associated with 90-day mortality. However, plasma markers of intestinal barrier integrity were not significantly different between the two groups. Forty-three ASVs identified from thrombus samples were significantly different in patients who died or survived at 90 days, as shown in the volcano plot (Fig. 3c). The LEfSe results suggested higher abundances of *Pseudomonadales* and Acinetobacter in the thrombi obtained from patients who died within 90 days (Fig. 3f).

**TABLE 3:** Comparisons in markers of inflammation and intestinal barrier integrity in patients who died or survived at 3 months^*a*^

Marker	Death group (*n* = 15)	Survival group (*n* = 80)	*P* value
WBC (10^9^/L)	11.85 (9.32–14.95)	9.83 (7.91–12.08)	0.024
CRP (mg/L)	13.66 (4.53–34.09)	8.36 (3.23–17.65)	0.368
IL-1β (pg/mL)	56.48 (34.22–187.37)	29.56 (20.95–47.13)	0.001
IL-6 (pg/mL)	35.24 (4.50–69.60)	5.10 (0–16.84)	0.001
TNF-α (pg/mL)	41.70 (33.70–69.26)	49.04 (40.27–57.56)	0.345
LPS (pg/mL)	43.21 (29.63–73.34)	50.38 (39.25–60.58)	0.571
LBP (μg/mL)	4.25 (3.42–6.85)	4.94 (3.67–5.91)	0.664
d-Lactate (mmol/L)	11.29 (7.73–12.34)	9.80 (7.34–12.50)	0.386

a Data are presented in medians (interquartile range). *P* values were determined by Mann-Whitney U tests. WBC, white blood cells; CRP, C-reactive protein; LPS, lipopolysaccharide; LBP, lipopolysaccharide-binding proteins.

Univariate Cox regression analyses revealed that prior stroke, preoperative NIHSS score, baseline white blood cell and neutrophil counts, baseline creatinine level, perioperative AE, and poststroke infection during hospitalization were associated with 90-day mortality. Of note, the abundance of *Pseudomonadales*, *Moraxellaceae*, and Acinetobacter was also associated with 90-day mortality (Table 4). In multivariate Cox regression analysis, Acinetobacter (hazard ratio [HR] 2.02; 95% confidence interval [CI], 1.20 to 3.41; *P* = 0.008) remained significantly associated with 90-day mortality after adjusting for other confounders.

**TABLE 4:** Univariate and multivariate Cox regression analyses for risk factors for 90-day mortality (*n* = 104)^*a*^

Parameter	Data for:			
	Univariate analysis		Multivariate analysis	
	HR (95% CI)	*P* value	aHR (95% CI)	*P* value
Age (yrs)	1.03 (0.99–1.07)	0.132		
Male sex	0.63 (0.24–1.64)	0.346		
Hypertension	0.67 (0.25–1.75)	0.411		
Diabetes mellitus	0.60 (0.20–1.83)	0.368		
Atrial fibrillation	2.60 (0.99–6.84)	0.053		
Prior stroke	4.07 (1.57–10.58)	0.004		NA
WBC (10^9^/L)	1.19 (1.07–1.33)	0.001		NA
NEU (10^9^/L)	1.22 (1.09–1.36)	0.0004		NA
Creatinine (μmol/L)	1.02 (1.00–1.03)	0.009		NA
CRP (mg/L)	1.01 (1.00–1.02)	0.108		
TC (mmol/L)	0.65 (0.41–1.04)	0.07		
LDL-C (mmol/L)	0.61 (0.33–1.14)	0.121		
Preoperative NIHSS score	1.09 (1.03–1.14)	0.001	1.13 (1.06–1.19)	0.0001
Onset-to-arrival time (min)	1.00 (1.00–1.00)	0.324		
Onset-to-reperfusion time (min)	1.00 (1.00–1.00)	0.126		
Perioperative AE	4.66 (1.64–13.23)	0.004	6.61 (2.06–21.15)	0.001
Poststroke infection	4.07 (1.17–14.16)	0.027		NA
Hospital stay (days)	0.86 (0.77–0.96)	0.008	0.88 (0.79–0.97)	0.011
Abundance of specific taxa in the thrombus (Ζ-score)				
Acinetobacter	1.98 (1.20–3.25)	0.007	2.02 (1.20–3.41)	0.008
*Moraxellaceae*	1.56 (1.04–2.36)	0.033		NA
*Pseudomonadales*	1.51 (1.02–2.23)	0.038		NA

a HR, hazard ratio; aHR, adjusted hazard ratio; 95% CI, 95% confidence interval; WBC, white blood cell; NEU, neutrophil; CRP, C-reactive protein; TC, total cholesterol; LDL-C, low-density lipoprotein cholesterol; NIHSS, National Institute of Health Stroke Scale; AE, adverse event; NA, not applicable.

There was no significant association of oral or fecal microbiota with 90-day mortality (Fig. S4c and e), but there were differential bacteria in the plasma samples of patients who died within 90 days (Fig. S5e), such as Acinetobacter, *Paracoccus*, and *Mesorhizobium*.

## DISCUSSION

The current study demonstrated the presence of bacteria in cerebral thrombi retrieved from LVO stroke patients, with bacterial DNA detected in 96.2% of the thrombus samples and clusters of bacterial signals seen with FISH. Compared with fecal and oral samples, the thrombus microbiota was mainly characterized by excessive enrichment of *Proteobacteria*, and traceability analysis indicated that thrombus bacteria mainly originated from plasma. Of note, there were differences in the microbial peculiarity in thrombi obtained from patients with CE and LAA stroke, with a higher concentration of bacterial DNA in CE strokes and different dominant bacteria and distribution patterns in the two groups. Higher abundances of Acinetobacter and *Enterobacteriaceae* in the thrombus were associated with a higher risk of perioperative AE within 48 h of admission. Moreover, an increased level of Acinetobacter in the thrombus was independently associated with a higher risk of death within 3 months. To the best of our knowledge, this is among the largest prospective cohorts to comprehensively characterize the microbial features of thrombus obtained by thrombectomy in LVO stroke patients and their associations with perioperative AE and 90-day mortality.

In this prospective study, 104 thrombi were obtained via mechanical thrombectomy within 24 h of symptom onset in AIS patients, and 96.2% of the thrombi were positive for bacterial DNA by 16S rRNA gene sequencing. Moreover, by means of FISH with a universal bacterial probe, we confirmed the presence of bacteria in the thrombus samples and observed clustering or freely distributed bacteria patterns. There may be doubts over possible contamination and false positivity in the findings. However, in the testing procedures, communications in and out of cells would stop after formalin fixation, and there would be no living bacteria in the tissue; then, the signals of possible contamination (if any) should be nonuniform after slicing, dehydration, and dyeing procedures. Yet the bright green fluorescence signals seen in the thrombus samples in FISH were mostly distributed in clusters inside and outside the cell, which were less likely to come from external contamination.

Previous studies had also confirmed the presence of bacteria in aspirated thrombi. In a previous study using qPCR to detect oral bacterial DNA in thrombi obtained from 75 stroke patients (22), 84.0% of the thrombus samples were bacteria positive, a bit lower than in our study (96.2%). In this previous study, DNA of Streptococcus species (representative oral microbe) was detected in 79% of the thrombus samples, comparable with that in the current study (73%). But the relative abundance of Streptococcus was not investigated in this previous study (22), which was low in the current study (1.1%), as revealed by next-generation sequencing. Another metagenomic analysis found more than 27 bacteria in middle cerebral artery thrombus samples from 4 stroke patients, with *Lactobacillus*, Staphylococcus, and *Stenotrophomonas* as the dominant bacteria (24), partially consistent with our findings. In our study, 182 genera and 542 ASVs had a relative abundance of >1.0% in the thrombi. The bacteria with the highest average abundance were Acinetobacter (12.8%) and *Stenotrophomonas* (5.3%), while *Lactobacillus* had an abundance of only 0.8%. In addition, Lactobacillus vaginalis, Bacillus cereus, and Kocuria marina were cultivated in the bacterial culture of the tissue fragment solution of thrombus aspirates in our study. Another study also confirmed the bacterial footprints in thrombus aspirates (14 positive in 124 patients) from patients with acute myocardial infarction by bacterial culture (26). It is possible that the majority of bacterial species in thrombi are commensal or low-grade pathogens around the human body, possibly invading the bloodstream from the oral tract, gut, or skin without leading to overt infection in immunocompetent hosts.

An innovation of this study is that oral, fecal, and isolated plasma samples of AIS patients were collected synchronously within 12 h of admission, aiming to more comprehensively illustrate the characteristics of the thrombus microbiome through multisite comparisons. Such cross-site comparisons could reduce the effects of between-individual variability in the microbiota on the findings, which also allow tracing of the origins of the thrombus microbiome (via the FEAST algorithm). For instance, a previous study reported shared operational taxonomic units (OTUs) between atherosclerotic plaques and the gut by using pyrosequencing of 16S rRNA genes of atherosclerotic plaque, oral, and gut samples of 15 patients (27). Consistent with common sense, this study showed less diverse microbiota in thrombus samples than oral and fecal samples, with significantly different microbial compositions. However, there were still 70 highly abundant ASVs in the thrombi overlapping with oral, fecal, and isolated plasma samples, suggesting that the source of the thrombus microbiome may be related to bacteria in these three regions. Bacteria in the thrombus and plasma samples were similar, and 46.7% of thrombus bacteria may originate from plasma samples based on the FEAST algorithm. Previous studies have also detected bacterial fragments in the plasma of healthy individuals (28) and ischemic stroke patients (29), of which several bacteria were culturable (28). However, the sources of the circulating bacteria were uncertain in these previous investigations. There were studies indicating possible translocation of bacteria from other body sites to the plasma (30, 31). For instance, gut bacteria such as Escherichia coli, Klebsiella pneumoniae, and Pseudomonas aeruginosa are common sources of bacteremia (32, 33), but not usually the direct cause of brain infection (34). Studies have associated cardiovascular diseases (35, 36) with increased translocation of intestinal bacteria, mainly through the epithelial mucosa of the intestine. In addition, oral bacteria (37–39) can also enter the blood with a compromised mechanical barrier of oral mucosa. Therefore, simultaneous collection and analysis of plasma, oral, and fecal samples with thrombus samples as in the current study could facilitate a better understanding of the potential sources of the microorganisms. However, oral and gut health status, which could significantly influence the bacterial signatures of oral and fecal samples and possible translocation of the bacteria, was not available in this cohort. This was a limitation of the current study, warranting further investigations.

In the current study, a majority of the bacteria present in the thrombus were from the phylum *Proteobacteria* (relative abundance, 73.3%), including 12 of the top 20 genera in the thrombus microbiome. An overabundance of *Proteobacteria* is generally considered a marker of gut dysbiosis (40) and a microbial signature of epithelial dysfunction (41). Previous studies have detected *Proteobacteria* in various body sites of humans and revealed a significant increase of *Proteobacteria* in atherosclerotic plaque (42). The present study revealed, for the first time, an overexpansion of *Proteobacteria* in thrombus in stroke patients, predicted to be a potential pathogenic bacterium by BugBase, while the underlying mechanisms remain unclear and warrant further research.

Our study also revealed different microbial characteristics of the thrombus in CE versus LAA strokes. In agreement with previous studies (24), *Lactobacillus* and *Chryseobacterium*, less common, non-lactose-fermenting bacteria that have emerged as important opportunistic pathogens (43), were significantly more abundant in thrombi from LAA stroke patients, while the *Veillonellaceae* family was more abundant in CE stroke patients. The findings suggested that bacteria, especially conditional pathogens, may be involved in thrombosis and LVO regardless of the stroke subtypes, of which the role may have been underestimated, but the associations between different bacteria and different stroke subtypes (e.g., CE or LAA) and the underlying mechanisms need further investigation. Moreover, the bacteria distribution patterns visualized with FISH in thrombi from CE and LAA strokes were different: a majority of the bacteria in thrombi from CE strokes were aggregated in leukocytes and on the surface of fibrin, while bacteria in thrombi from LAA strokes were mostly freely distributed on the surfaces of red blood cells.

Bacteria in systemic circulation may participate in the thrombosis process under certain stimulations. Previous studies have demonstrated that the presence of bacteria is conducive to thrombosis even in the absence of *in situ* atherosclerosis. Once bacteria gather in clusters, the specific spatial localization of *Bacillus* may affect the coagulation process and directly activate coagulation within minutes (44). In addition, some bacterial species, such as Staphylococcus aureus, could secrete procoagulant factors and promote plasma clotting to avoid deadly attack from the immune system (45). Our study showed that both *Bacillus* and Staphylococcus were present in aspirated thrombus from LVO stroke patients, with relative abundances of 2.1% and 1.2%, respectively. Strikingly, based on the prediction of BugBase, the thrombus community was dominated by Gram-negative bacteria, and the biofilm formation ability was significantly enhanced compared to that in other samples. Therefore, bacteria in the thrombus may play a critical role in promoting blood coagulation.

Early AEs (hemorrhagic transformation, etc.) after mechanical thrombectomy are associated with poor prognosis in LVO stroke patients (46–49). In our study, the levels of Acinetobacter and *Enterobacteriaceae* (both opportunistic pathogens) in thrombus samples were significantly higher in patients with perioperative AEs within 48 h of admission than in those without. Moreover, a higher abundance of Acinetobacter in the thrombus was significantly associated with a higher risk of 3-month mortality in LVO stroke patients, independent of other confounding factors. Acinetobacter species are omnipresent organisms that are widely distributed in nature and contribute to a range of nosocomial infections (50, 51). Enrichment of *Enterobacteriaceae* in the gut is associated with a higher risk of stroke (52) and is an independent predictor of poor outcomes after stroke (3). In addition, we also found increased abundance of Acinetobacter in plasma samples of patients who developed perioperative AE or died within 90 days, which corroborated the associations between thrombus microbiota and the clinical outcomes. A better understanding of the mechanisms underlying the associations between these bacteria and worse outcomes after stroke and targeted regulation of the microbiome in plasma and/or thrombus may help prevent thrombosis, reduce stroke risks, and improve prognosis of stroke patients.

The current study had limitations. First, although the current study was among the largest cohorts of this kind, we had not conducted sample size and power estimation before initiation of this exploratory, observational study. However, the current findings could provide a basis for power estimation for future prospective studies in Chinese and other populations. Second, a universal bacterial probe, rather than specific markers of bacterial strains, was used in FISH, so it was impossible to tell whether the clusters of bacteria were from a single strain or mixed coenobium. In addition, we did not assess bacterial viability of each thrombus in the current study due to the difficulty in collecting enough thrombus fragments for bacterial culture and sequencing synchronously. Future studies using specific bacterial staining and bacterial viability tests may help reveal the role of specific bacteria in thrombosis and prognosis after stroke. Moreover, findings from this clinical observational study need to be verified in animal models and at cellular levels in future studies.

### Conclusions.

Our study confirmed the existence of bacteria in a majority of thrombi retrieved from stroke patients with LVO and shed light on the characteristics of the thrombus microbiome. It also revealed possible differences in the bacterial concentrations, dominant bacteria, and distribution patterns in thrombi obtained from CE and LAA strokes. Moreover, a higher abundance of opportunistic pathogens in the thrombus was closely associated with higher risks of perioperative AEs and 90-day mortality after mechanical thrombectomy in LVO stroke. Future larger-scale studies with bacterial culture, specific bacterial staining, and receptor expression detection are needed to verify the role of microbiota in thrombosis, stroke occurrence and prognosis, and the underlying mechanisms.

## MATERIALS AND METHODS

### Study subjects and sample collection.

**FIG 5:**
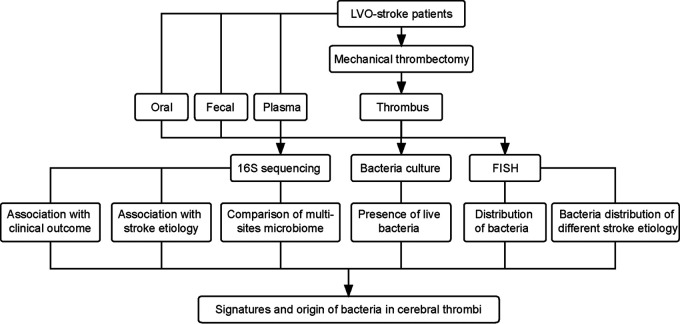
Flowchart of the study. Abbreviations: LVO, large vessel occlusion; FISH, fluorescent *in situ* hybridization.

This was a prospective cohort study. We recruited AIS patients admitted to the Acute Stroke Unit of the First Affiliated Hospital of Jinan University between June 2019 and June 2020, a university-affiliated academic hospital in Guangzhou, China, and the patients were treated by mechanical thrombectomy within 24 h of symptom onset. The inclusion criteria were (i) adult AIS patients (>18 years) treated with mechanical thrombectomy with retrieval of the thrombus aspirates, (ii) availability of the thrombus for bacterial and histopathological analyses, (iii) the stroke etiology was cardioembolism (CE) or large-artery atherosclerosis (LAA) according to the Trial of ORG 10172 in Acute Stroke Treatment (TOAST) (53) classification, (iv) no infectious disease or treatment with antibiotics or probiotics within 1 month prior to the index stroke or before sample collection, and (v) all patients had successful revascularization with the modified Thrombolysis in Cerebral Infarction (mTICI) score of ≥2b/3 (54). Other causes of LVO, such as tumor embolus, were excluded from the study. Two experienced neurologists examined all patients upon arrival at the hospital and evaluated the eligibility for mechanical thrombectomy according to the latest clinical guidelines (55) but did not interfere with the current study. Thrombus, fecal, oral, and plasma samples (Fig. 5; Fig. 1a) were collected from each recruited patient within 12 h after admission and transferred to a −80°C freezer for further 16S sequencing. Histories of smoking, drinking, and common diseases; stroke severity by NIH Stroke Scale (NIHSS); hospital stay length; and other relevant information were collected.

The thrombus was removed during surgery, rinsed with 0.9% sterile normal saline, and placed directly into sterile tubes (catalog no. CY-95000-P1; HCY Technology, China) for qPCR analysis under strict aseptic conditions. A histological sample was placed in another tube with 3.7% formaldehyde if the thrombus was of adequate size. All of the thrombus collection and preparation processes were carried out under sterile conditions, and the thrombus did not come into contact with any potential contaminant. Blank controls were obtained in 4 cases via swabs before thrombus collection from collection tubes and anything that could be in contact with the thrombus on the operation table.

For oral samples, the buccal mucosa and tooth surface on both sides of the oral cavity were wiped with sterile swabs and put into the matching sterile tube. The presence of gingival bleeding or acute periodontal disease was recorded. For fecal samples, rectal swabs instead of stool samples were used in this study for microbiota analyses, as AIS patients were generally under sedation within 24 h after endovascular treatment. A sterile swab was inserted into the anus for 4 to 7 cm, rotated several times, and then taken out and put into a sterile tube. In addition, 4 mL of blood was drawn from the median cubital vein in each patient and centrifuged at low temperature (3,000 rpm/min, 4°C, 10 min), and the upper plasma was placed in a sterile tube.

Perioperative adverse events (AEs) within 48 h of admission were recorded, including new neurological symptoms with new ischemic lesion(s) confirmed in diffusion-weighted magnetic resonance (MR) imaging, hemorrhagic transformation, and cerebral herniation. Hemorrhagic transformation was defined as hemorrhagic infarction or parenchymal hematoma as confirmed with brain computed tomography (CT) according to the European Cooperative Acute Stroke Study (ECASS II) criteria (56). Poststroke infection during hospitalization was also recorded, including respiratory infection, urinary infection, and septicemia. All patients were followed up for 90 days, with a primary outcome of death within 90 days.

### Ethics statement.

The study was approved by the Medical Ethics Committee of the First Affiliated Hospital of Jinan University (no. KY-2020-030) and conducted in accordance with the Declaration of Helsinki. Written informed consent was obtained from all study participants or their legal representatives.

### DNA extraction and 16S sequencing.

Bacterial DNA was extracted from the samples, enriched by PCR amplification, and identified by 16S sequencing with the following steps. First, bacterial DNA was extracted from samples using a commercially available bacterial DNA extraction minikit (MabioDNB361B; Mabio, China). Blank extractions (as reagent controls) were also processed and used to identify potential contamination. The V3-V4 variable regions of 16S rRNA genes were then amplified by PCR using Illumina-tagged primers 338F (5′-ACTCCTACGGGAGGCAGCA-3′) and 806R (5′-GGACTACHVGGGTWTCTAAT-3′). Three replicates of the PCR products in the same sample were mixed, and the PCR instrument was a Bio-Rad S1000 (Bio-Rad Laboratories, CA). PCR products were evaluated by 1.5% agarose gel electrophoresis, and visible products 470 bp (with barcode) in size were used in further experiments. The PCR products were mixed in equimolar ratios, calculated by GeneTools analysis software (version 4.03.05.0; SynGene), and then purified using an EZNA gel extraction kit (Omega, USA). Sequencing libraries were set up using the NEBNext Ultra DNA library prep kit for Illumina (New England Biolabs, USA), and the index codes were added. The Qubit 2.0 fluorometer (Thermo Scientific) and Agilent Bioanalyzer 2100 system were used to evaluate the library quality. Finally, the library was sequenced on an Illumina HiSeq 2500 platform, and 250-bp paired-end reads were obtained.

### Bioinformatics analyses.

Bioinformatics analyses were performed to compare the bacterial signatures of different samples and to track the sources of bacteria in thrombus samples. The 16S rRNA gene sequences were processed using VSEARCH 2.15.1 (57), USEARCH 10.0.240 (58), and in-house scripts (59). Based on the high-confidence 16S representative sequences, an amplicon sequence variant (ASV) table was created according to VSEARCH (–usearch_global). Linear discriminant analysis (LDA) coupled with effect size measurement (LEfSe) was used to measure differentially abundant taxa across groups using the default parameters (60). Based on high-quality sequences, BugBase (61) was used to infer and compare organism-level microbiome phenotypes among different samples to better understand the possible function of the bacteria in the thrombus. FEAST was performed to track the microbial source based on the ASV data following protocols provided by the authors of the R package (62). Contribution from each source was calculated and represented as a percentage. Gut microbiota alpha diversity was calculated by the Shannon index with the vegan package, and between-sample differences were tested with Kruskal-Wallis tests, with subsequent pairwise comparisons adjusted by Bonferroni correction. The microbial beta diversity was compared using Bray-Curtis dissimilarity (unconstrained principal coordinate analysis [PCoA]), and significance was determined by the Adonis test. The relative abundance of dominant taxa in each patient or each group by the outcomes is shown in stack bar plots using the R package ggplot2.

### Bacterial culture.

We conducted bacterial culture of thrombus aspirates in eight additional cases (MoonBiotech Co., Ltd., Guangzhou, China) to confirm the presence of live bacteria. In short, fresh thrombus was rinsed with 0.9% normal saline, placed in sterile phosphate-buffered saline (PBS) solution immediately, and transported to the laboratory at low temperature (4°C) within 2 h. After washing with sterile water and surface disinfection, thrombus tissue was mechanically broken, and the broken solution was coated in the separation medium for culture. The bacterial colonies, if any, were purified and identified.

### Fluorescent *in situ* hybridization.

FISH of tissue sections was conducted in patients with adequate thrombus tissue to further confirm the presence of bacteria and visualize their distribution in the thrombus (Fig. 3a). The blood attached to the thrombus was rinsed with 0.9% normal saline, and the thrombus was fixed with 3.7% formaldehyde. The specimens were dewaxed with xylene three times, dehydrated in an ethanol series (98.0%, 85.0%, and 70.0%), and then washed two times in PBS (each step for 5 min). The bacterial signals were labeled by the universal bacterial probe EUB338 (1:20; catalog no. FB-0010B; HYCX, China), and the slides were processed with a FISH kit (catalog no. D-0016; Exon Biotechnology, China). Briefly, sections were immersed in blocking solution at 55°C for 2 h and then hybridized overnight at 37°C with EUB338 diluted with hybridization buffer in a humid chamber. The unbound probe was subsequently removed by incubation in wash buffer. Nuclei were stained with 4,6-diamidino-2-phenylindole (DAPI) for 15 min. The slides were air-dried and analyzed by fluorescence microscopy (Zeiss Scope.A1; oil immersion lens; magnification, ×1,000), and images were processed using ImageJ software.

### Enzyme-linked immunosorbent assay.

Isolated plasma collected from AIS patients within 12 h of admission was assayed for inflammatory cytokines and markers of intestinal barrier integrity by using ELISA kits. Tumor necrosis factor-α (TNF-α; catalog no. CSB-E04740h), interleukin-1β (IL-1β; catalog no. CSB-E08053h), interleukin-6 (IL-6; catalog no. CSB-E04638h), lipopolysaccharide (LPS; catalog no. CSB-E09945h), and lipopolysaccharide-binding protein (LBP; catalog no. CSB-E09629h) were assayed by ELISA kits (Cusabio, China). d-Lactate (Catalog no. CAK1177) was analyzed using a kit and protocol from Cohesion Bioscience, China.

### Other statistical analyses.

Statistical analyses were performed using R 4.0.3 and SPSS 27.0 (IBM, USA). Continuous variables were expressed with medians (interquartile range [IQR]) and compared between groups using Mann-Whitney U tests or Kruskal-Wallis tests. Subsequent pairwise comparisons were adjusted by Bonferroni correction. Medians with quartiles of DNA concentrations, Shannon indices, microbiome phenotypes, and bacteria distributions were plotted in boxplots. Other statistics used in bioinformatics analysis are described above.

Univariate Cox regression analyses were conducted to identify potential factors associated with 90-day mortality, such as demographics (age and sex), medical history, and comorbidities (diabetes, hypertension, atrial fibrillation, prior stroke), plasma markers (white blood cell count, neutrophil count, creatinine, C-reactive protein, total cholesterol, and low-density lipoprotein cholesterol), stroke severity by preoperative NIHSS, onset-to-arrival and onset-to-reperfusion time, Ζ-scores of specific taxon abundances (63), perioperative AEs, poststroke infection, and length of hospital stay. Variables with *P* values of <0.05 in univariate analyses were further analyzed in multivariate Cox regression with the forward (LR) method. Two-sided *P* values of <0.05 were considered of statistical significance.

### Data availability.

Data generated or analyzed during this study are included in this article and the supplemental files. Sequence data are available from the National Center for Biotechnology Information (NCBI) Sequence Read Archive (SRA) database (https://www.ncbi.nlm.nih.gov/; SRA accession no. PRJNA764274) after publication of the article. Other data and codes that support the findings of this study are available from the corresponding author upon reasonable request.
