# Supplementary figures and images for: Bacterial Signatures of Cerebral Thrombi in Large Vessel Occlusion Stroke
Source: mBio. 2022 Jun 21;13(4):e01085-22. doi: 10.1128/mbio.01085-22 (PMC9426487; doi:10.1128/mbio.01085-22)

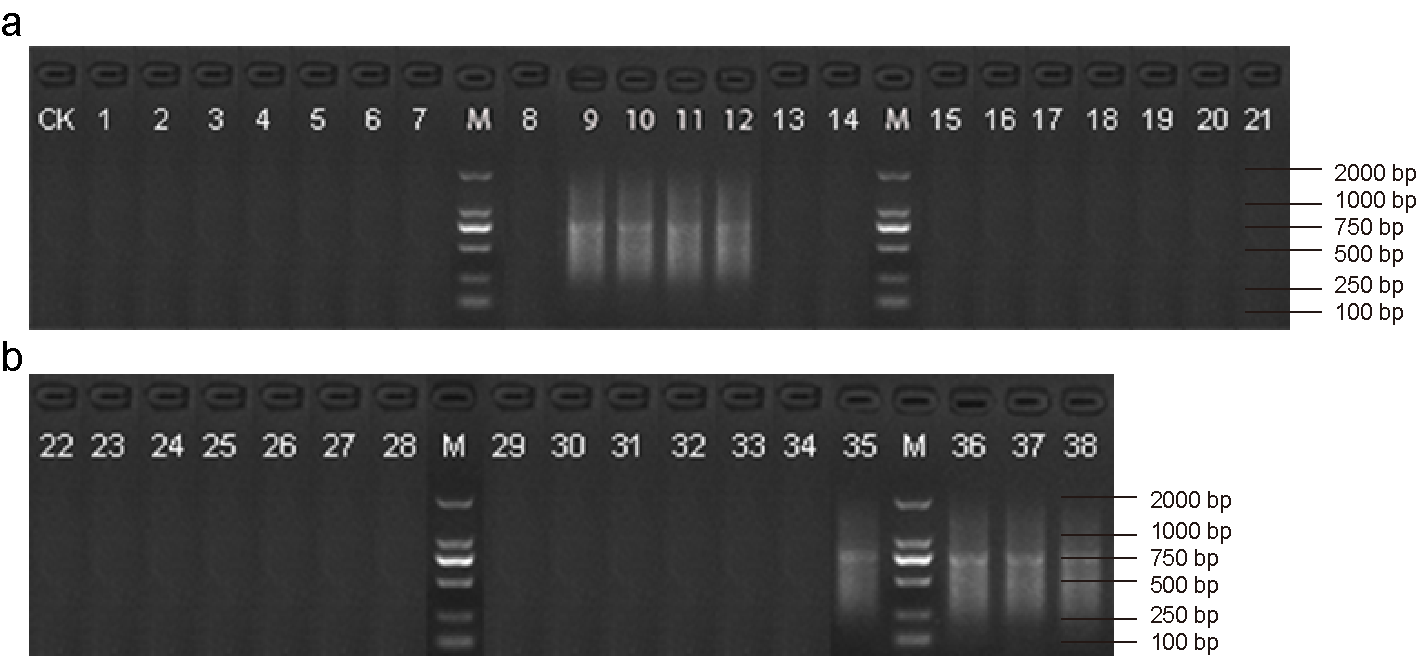

Supplement: FIG S1 [file mbio.01085-22-s0001.tif]

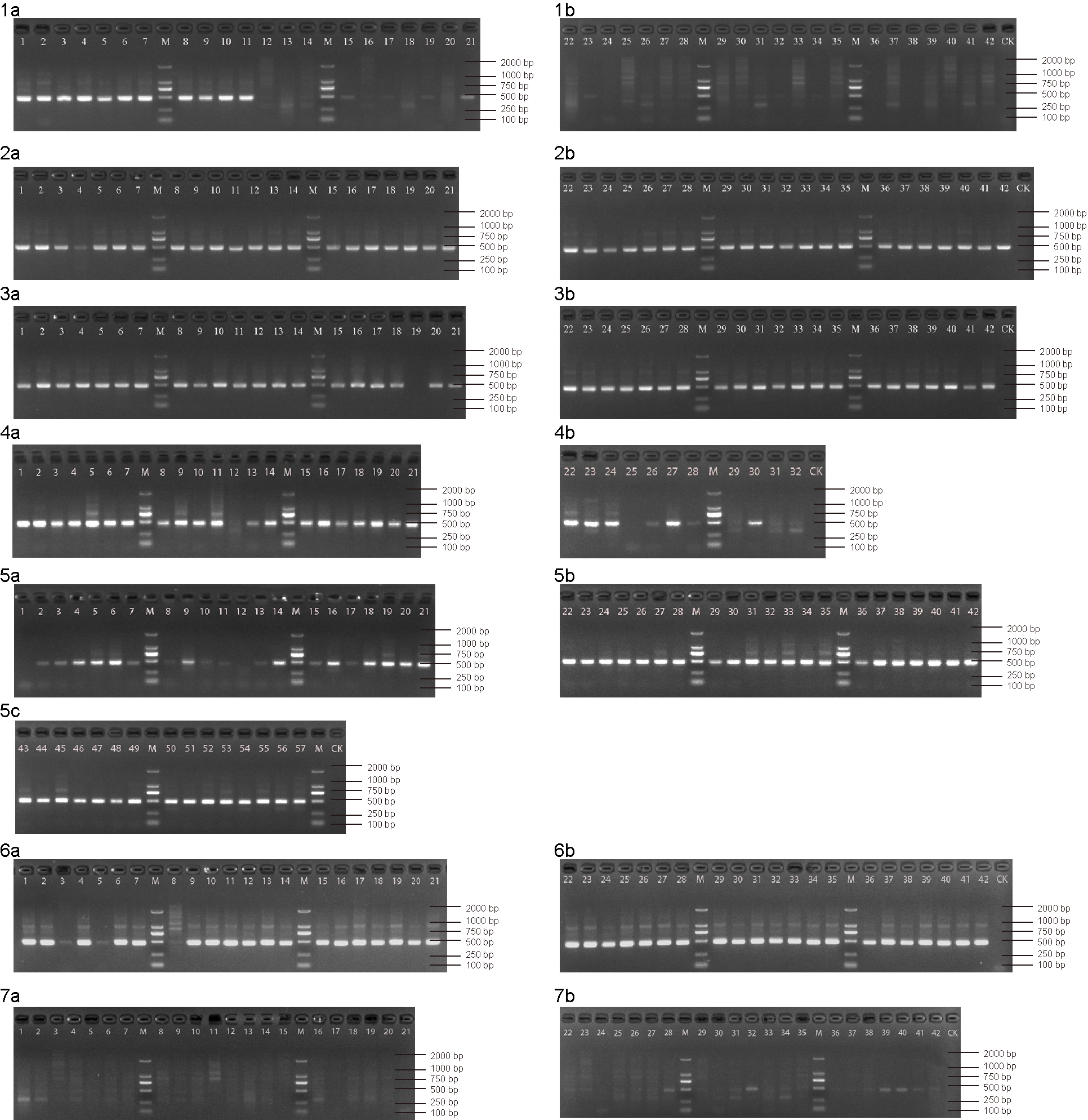

Supplement: FIG S2 [file mbio.01085-22-s0002.tif]

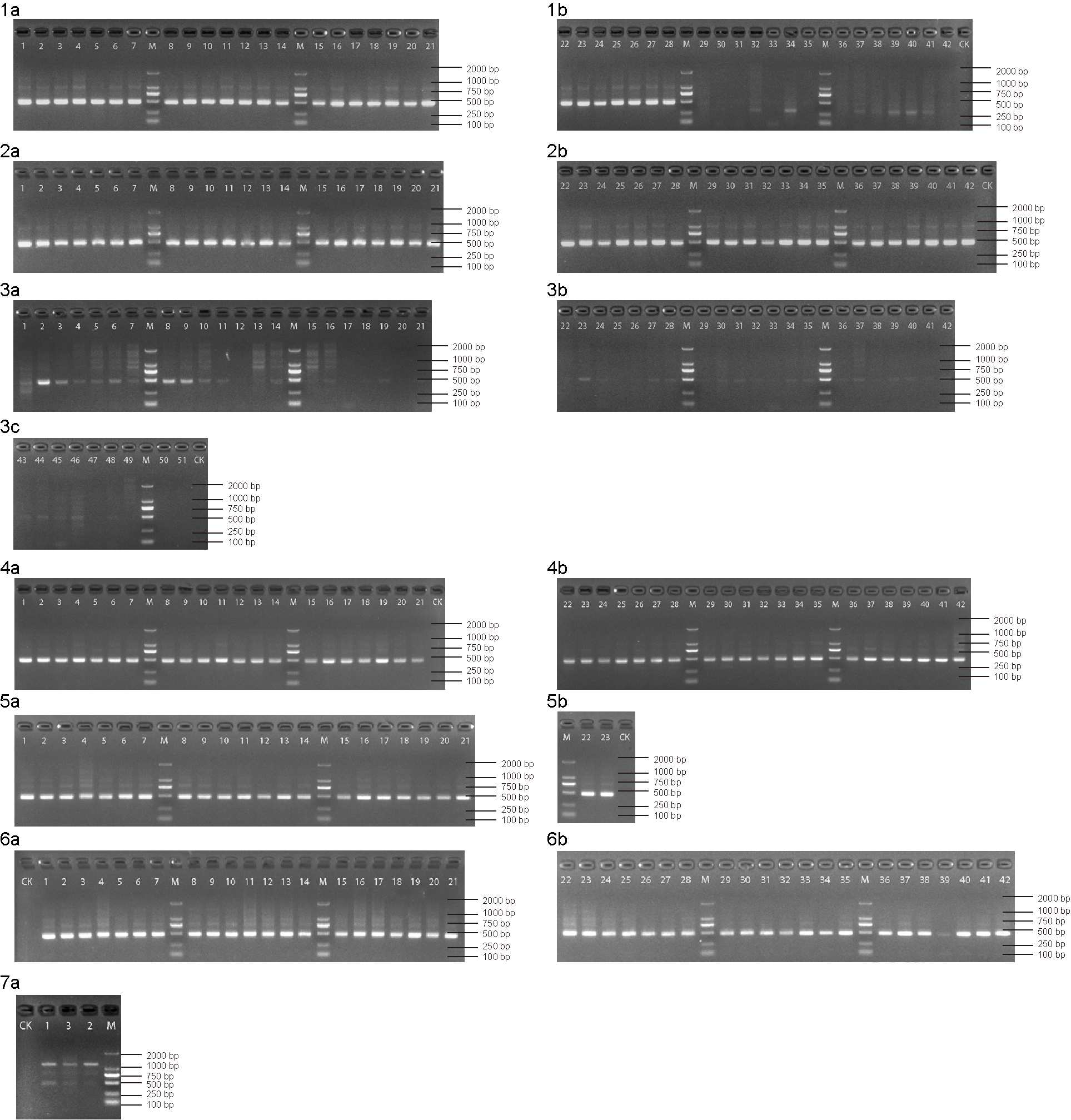

Supplement: FIG S3 [file mbio.01085-22-s0003.tif]

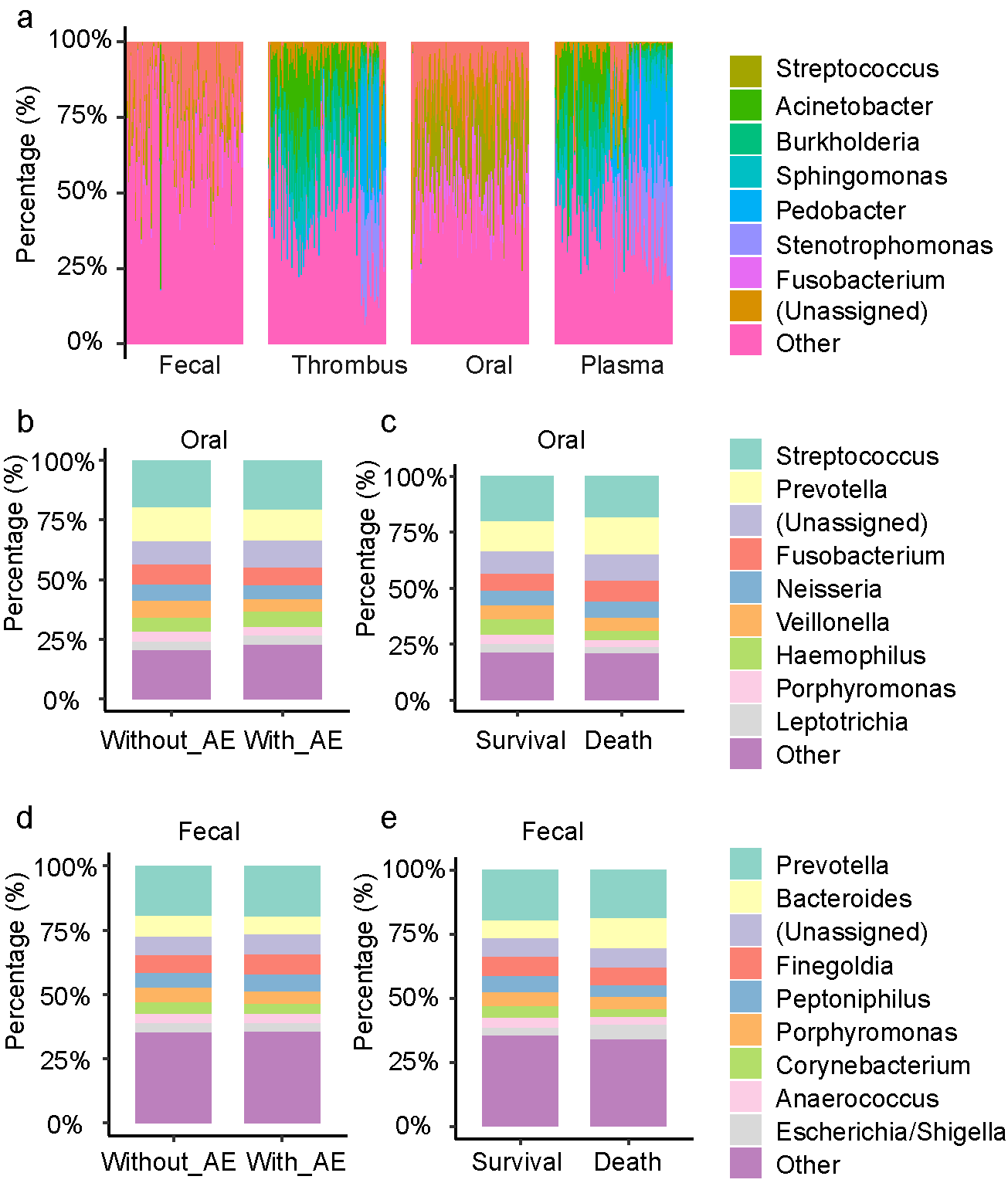

Supplement: FIG S4 [file mbio.01085-22-s0004.tif]

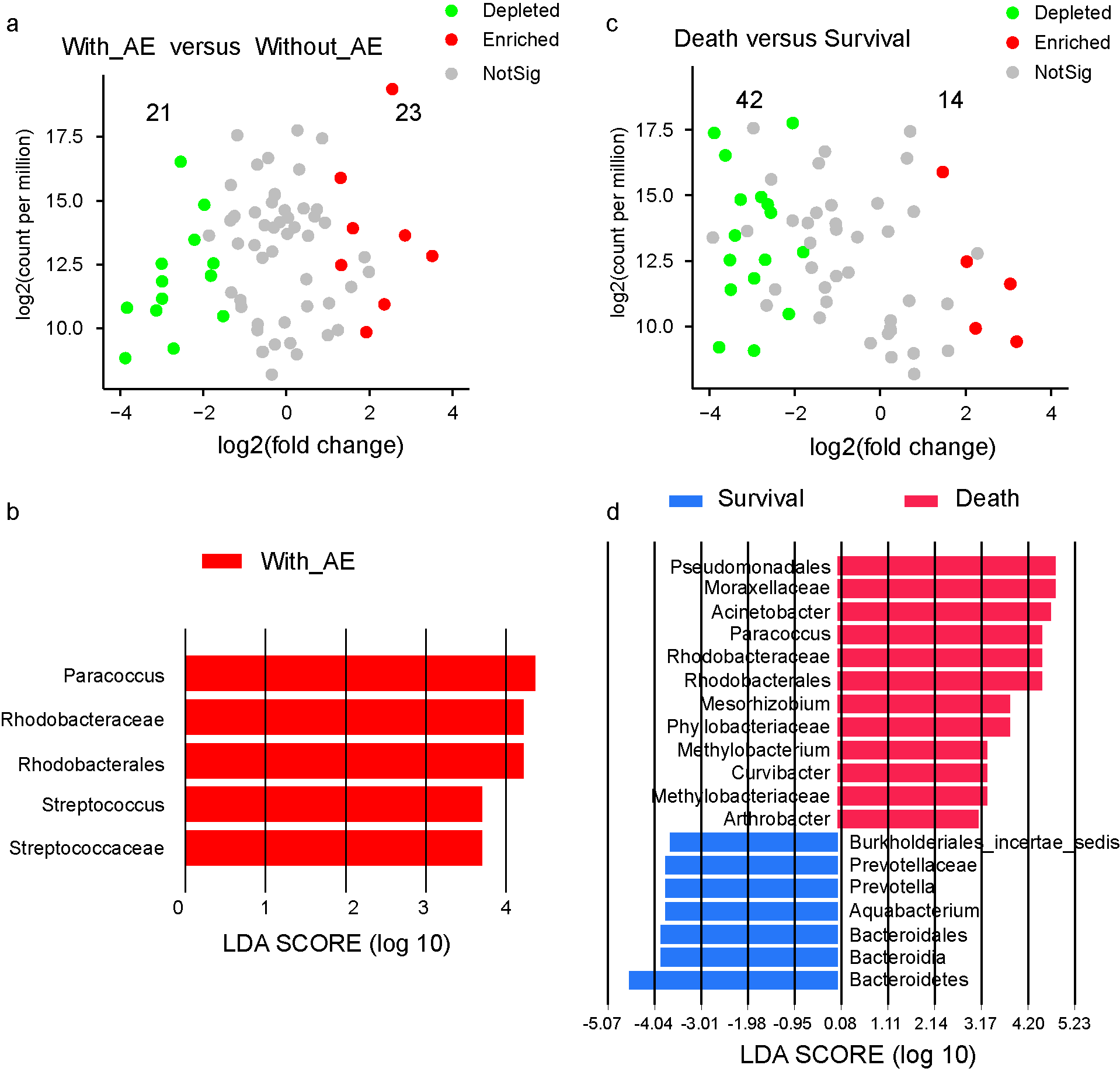

Supplement: FIG S5 [file mbio.01085-22-s0005.tif]
